# 1-(2-Hy­droxy­eth­yl)-1′-methyl-4′-(naph­thal­en-1-yl)-1′′,2′′,3′′,4′′-tetra­hydro­dispiro­[indoline-3,2′-pyrrolidine-3′,2′′-naphthalene]-2,1′′-dione

**DOI:** 10.1107/S1600536812006617

**Published:** 2012-02-24

**Authors:** S. Selvanayagam, B. Sridhar, P. Saravanan, R. Raghunathan

**Affiliations:** a Department of Physics, Kalasalingam University, Krishnankoil 626 126, India; b Laboratory of X-ray Crystallography, Indian Institute of Chemical Technology, Hyderabad 500 007, India; c Department of Organic Chemistry, University of Madras, Guindy Campus, Chennai 600 025, India

## Abstract

In the title compound, C_33_H_30_N_2_O_3_, the pyrrolidine ring adopts an envelope conformation in which the H atom attached the an *ortho*-C atom deviates from the plane, whereas the cyclo­hexa­none ring in the tetra­hydro­naphthalene fused-ring system adopts a sofa conformation. The oxindoline ring system is almost perpendicular with respect to the mean plane of the pyrrolidine ring, with a dihedral angle of 89.0 (1)°. Five intra­molecular C—H⋯O close contacts are observed. In the crystal, mol­ecules associate *via* O—H⋯O hydrogen bonds, forming *R*_2_^2^(14) dimers. In addition, there are weak C—H⋯π inter­actions.

## Related literature

For general background to pyrrolidine derivatives, see: Sundar *et al.* (2011 ▶); Crooks & Sommerville (1982 ▶); Stylianakis *et al.* (2003 ▶). For a related structure, see: Selvanayagam, Ravikumar *et al.* (2011 ▶); Selvanayagam, Sridhar *et al.* (2011 ▶). For ring-puckering parameters, see: Cremer & Pople (1975 ▶); Nardelli (1983 ▶).
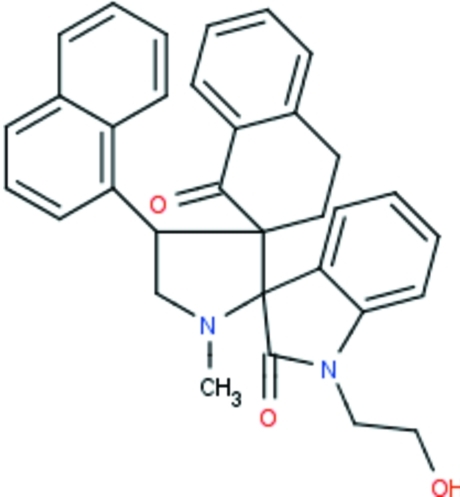


## Experimental

### 

#### Crystal data

C_33_H_30_N_2_O_3_
*M* *_r_* = 502.59
Monoclinic, 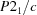

*a* = 12.0236 (18) Å
*b* = 14.054 (2) Å
*c* = 15.950 (2) Å
β = 107.796 (2)°
*V* = 2566.2 (7) Å^3^
*Z* = 4
Mo *K*α radiation
μ = 0.08 mm^−1^
*T* = 292 K
0.22 × 0.20 × 0.18 mm

#### Data collection

Bruker SMART APEX CCD area-detector diffractometer
29561 measured reflections
6100 independent reflections
4956 reflections with *I* > 2σ(*I*)
*R*_int_ = 0.021

#### Refinement

*R*[*F* ^2^ > 2σ(*F* ^2^)] = 0.044
*wR*(*F* ^2^) = 0.130
*S* = 1.02
6100 reflections
345 parameters
H-atom parameters constrained
Δρ_max_ = 0.27 e Å^−3^
Δρ_min_ = −0.16 e Å^−3^

### 

Data collection: *SMART* (Bruker, 2001 ▶); cell refinement: *SAINT* (Bruker, 2001 ▶); data reduction: *SAINT*; program(s) used to solve structure: *SHELXS97* (Sheldrick, 2008 ▶); program(s) used to refine structure: *SHELXL97* (Sheldrick, 2008 ▶); molecular graphics: *ORTEP-3* (Farrugia, 1997 ▶) and *PLATON* (Spek, 2009 ▶)’; software used to prepare material for publication: *SHELXL97* and *PLATON*.

## Supplementary Material

Crystal structure: contains datablock(s) I, global. DOI: 10.1107/S1600536812006617/bt5819sup1.cif

Structure factors: contains datablock(s) I. DOI: 10.1107/S1600536812006617/bt5819Isup2.hkl

Additional supplementary materials:  crystallographic information; 3D view; checkCIF report

## Figures and Tables

**Table 1:** Hydrogen-bond geometry (Å, °) *Cg*1 is the centroid of the C10–C15 benzene ring.

*D*—H⋯*A*	*D*—H	H⋯*A*	*D*⋯*A*	*D*—H⋯*A*
C26—H26⋯O1	0.93	2.54	3.198 (2)	128
C24—H24*A*⋯O2	0.97	2.42	3.098 (2)	127
C14—H14⋯O1	0.93	2.59	3.394 (2)	145
C2—H2⋯O1	0.98	2.21	2.764 (2)	115
C1—H1*B*⋯O2	0.97	2.40	3.020 (2)	121
O3—H3⋯O2^i^	0.82	2.03	2.830 (1)	164
C20—H20⋯*Cg*1^ii^	0.93	2.71	3.603 (2)	161

Symmetry codes: (i) 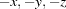; (ii) 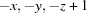.

**Fig. 1.**
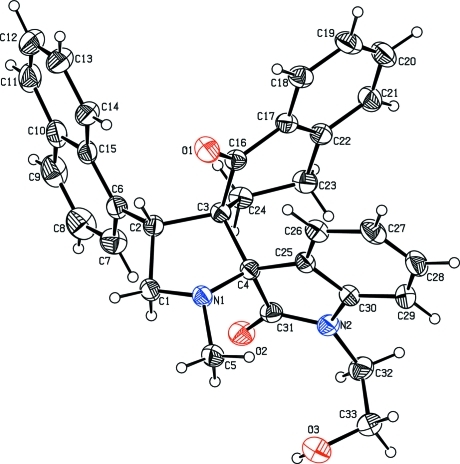
The molecular structure of the title compound, showing the atom-numbering scheme. Displacement ellipsoids are drawn at the 30% probability level

**Fig. 2.**
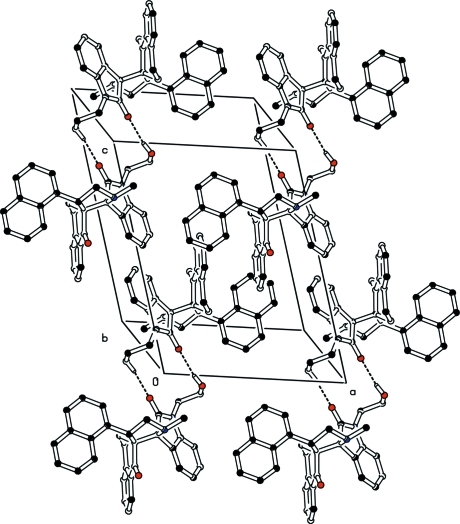
Molecular packing of the title compound, viewed down the b axis; H-bonds are shown as dashed lines forms a R22(14) dimers in unit cell. For the sake of clarity, H atoms, not involved in hydrogen bonds, have been omitted

**Fig. 3.**
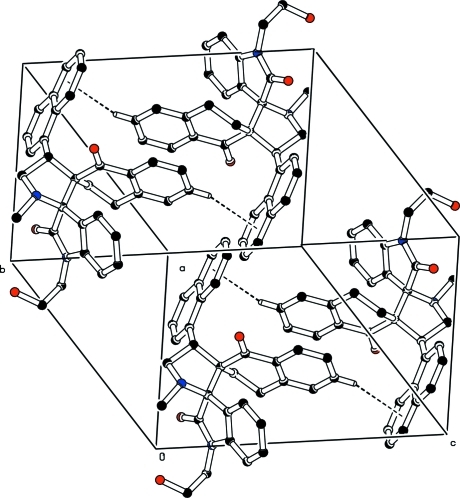
Molecular packing of the title compound showing C—H···π interactions in unit cell. For the sake of clarity, H atoms, not involved in hydrogen bonds, have been omitted

## supplementary crystallographic information

### Comment

Spiro-pyrrolidine ring system is a structural motif in many biologically important and pharmacologically relevant alkaloids. These derivatives are used as antimicrobial and antitumour agents (Sundar *et al.*, 2011). These derivatives possess analgesic (Crooks & Sommerville, 1982) and anti-influenza virus (Stylianakis *et al.*, 2003) activities. In view of these importance and continuation of our work on the crystal structure analyis of spiro-pyrrolidine derivatives, we have undertaken the crystal structure determination of the title compound, and the results are presented here.

The X-ray study confirmed the molecular structure and atomic connectivity for (I), as illustrated in Fig. 1. The geometry of pyrrolidine, tetrahydro naphthalene and naphthyl group systems are comparable with the related reported structure (Selvanayagam, Ravikumar *et al.*, 2011; Selvanayagam, Sridhar *et al.*, 2011).

The sum of the angles at N1 of the pyrrolidine ring [335.5°] and N2 of the oxindole ring [359.7°] are in accordance with sp^3^ and sp^2^ hybridizations. The short contacts H1B···H7 (2.2 Å) and H2···H14 (1.9 Å) result in substantial widening of the C6—C7—C8 and C14—C15—C6 bond angles [121.8 (2)° and 123.7 (1)°, respectively].

Pyrrolidine ring adopts an envelope conformation, with puckering parameters q_2_ = 0.431 (1) Å and φ = 11.8 (1) °, and with atom C1 deviating 0.606 (1) Å from the least-squares plane passing through the remaining four atoms (N1/C2-C4) of that ring (Cremer & Pople, 1975). The cyclohexanone ring in the tetrahydro naphthaline ring system has a sofa conformation with the lowest asymmetry parameters of ΔC_2_(C3-C24) = 0.085 (1)° (Nardelli, 1983). The naphthalene ring system is oriented with a dihedral angles of 88.5 (1) and 41.8 (1)°, respectively with respect to the best plane of pyrrolidine ring and oxindole ring systems.

The molecular structure is influenced by an intramolecular C—H···O close contacts. Atom O1 acts as a trifurcated acceptor for three intramolecular C—H···O contacts. In the molecular packing, O—H···O hydrogen bonds involving atoms O3 and O2 link inversion-related molecules to form R_2_^2^ (14) graph-set dimer (Fig. 2 and Table 1). In addition to this intermolecular C—H···π interactions are formed such that atom H20 is 2.71 Å from the centroid of the phenyl ring (C10-C15) at (-x,-y,1-z), with C20—H20··· centroid angle of 161° and C20···centroid distance of 3.603 (2) Å (Fig. 3).

### Experimental

To a mixture of 1-(2-hydroxyethyl)indoline-2,3-dione (1mmol), sarcosine (1mmol) and 2-naphthalidene-1,2,3,4-tetrahydronaphthalene-1-one (1mmol) was added and heated under reflux in methanol (20ml) until the disappearance of the starting materials as evidenced by TLC. The solvent was removed under vacuo and the crude product was subjected to column chromatography using petroleum ether-ethyl acetate eluent. Single crystals were grown by slow evaporation from methanol.

### Refinement

H atoms were placed in idealized positions and allowed to ride on their parent atoms, with C—H distances of 0.93-0.98 Å and O—H distance of 0.82 Å, and Uiso(H) = 1.5U_eq_(C) for methyl H and Uiso(H) = 1.2U_eq_(C or O) for all other H atoms.

### Crystal data

**Table:**

C_33_H_30_N_2_O_3_	*F*(000) = 1064
*M**_r_* = 502.59	*D*_x_ = 1.301 Mg m^−^^3^
Monoclinic, *P*2_1_/*c*	Mo *K*α radiation, λ = 0.71073 Å
Hall symbol: -P 2ybc	Cell parameters from 18685 reflections
*a* = 12.0236 (18) Å	θ = 2.2–27.8°
*b* = 14.054 (2) Å	µ = 0.08 mm^−^^1^
*c* = 15.950 (2) Å	*T* = 292 K
β = 107.796 (2)°	Block, colourless
*V* = 2566.2 (7) Å^3^	0.22 × 0.20 × 0.18 mm
*Z* = 4	

### Data collection

**Table:**

Bruker SMART APEX CCD area-detector diffractometer	4956 reflections with *I* > 2σ(*I*)
Radiation source: fine-focus sealed tube	*R*_int_ = 0.021
Graphite monochromator	θ_max_ = 28.0°, θ_min_ = 1.8°
ω scans	*h* = −15→15
29561 measured reflections	*k* = −18→18
6100 independent reflections	*l* = −21→20

### Refinement

**Table:**

Refinement on *F*^2^	Primary atom site location: structure-invariant direct methods
Least-squares matrix: full	Secondary atom site location: difference Fourier map
*R*[*F*^2^ > 2σ(*F*^2^)] = 0.044	Hydrogen site location: inferred from neighbouring sites
*wR*(*F*^2^) = 0.130	H-atom parameters constrained
*S* = 1.02	*w* = 1/[σ^2^(*F*_o_^2^) + (0.0763*P*)^2^ + 0.3393*P*] where *P* = (*F*_o_^2^ + 2*F*_c_^2^)/3
6100 reflections	(Δ/σ)_max_ < 0.001
345 parameters	Δρ_max_ = 0.27 e Å^−^^3^
0 restraints	Δρ_min_ = −0.16 e Å^−^^3^

### Special details

**Table:**

Geometry. All esds (except the esd in the dihedral angle between two l.s. planes) are estimated using the full covariance matrix. The cell esds are taken into account individually in the estimation of esds in distances, angles and torsion angles; correlations between esds in cell parameters are only used when they are defined by crystal symmetry. An approximate (isotropic) treatment of cell esds is used for estimating esds involving l.s. planes.
Refinement. Refinement of F^2^ against ALL reflections. The weighted R-factor wR and goodness of fit S are based on F^2^, conventional R-factors R are based on F, with F set to zero for negative F^2^. The threshold expression of F^2^ > 2sigma(F^2^) is used only for calculating R-factors(gt) etc. and is not relevant to the choice of reflections for refinement. R-factors based on F^2^ are statistically about twice as large as those based on F, and R- factors based on ALL data will be even larger.

### Fractional atomic coordinates and isotropic or equivalent isotropic displacement parameters (Å^2^)

**Table:**

	*x*	*y*	*z*	*U*_iso_*/*U*_eq_	
N1	0.10384 (8)	0.22419 (7)	0.19534 (6)	0.0402 (2)	
N2	0.00850 (9)	−0.00310 (7)	0.19134 (7)	0.0459 (2)	
O1	0.30700 (9)	0.24467 (6)	0.39617 (6)	0.0558 (2)	
O2	0.11658 (9)	0.03889 (7)	0.10210 (6)	0.0555 (2)	
O3	−0.19054 (11)	0.03227 (8)	0.03809 (7)	0.0652 (3)	
H3	−0.1606	0.0206	−0.0006	0.098*	
C1	0.19263 (10)	0.24133 (9)	0.15200 (8)	0.0434 (3)	
H1A	0.1927	0.3073	0.1341	0.052*	
H1B	0.1812	0.2007	0.1009	0.052*	
C2	0.30466 (10)	0.21616 (8)	0.22418 (7)	0.0399 (2)	
H2	0.3234	0.2710	0.2640	0.048*	
C3	0.26925 (9)	0.13307 (7)	0.27700 (7)	0.0365 (2)	
C4	0.13143 (10)	0.13071 (8)	0.23768 (7)	0.0369 (2)	
C5	−0.01552 (11)	0.23600 (10)	0.13839 (9)	0.0523 (3)	
H5A	−0.0279	0.3010	0.1194	0.078*	
H5B	−0.0689	0.2195	0.1702	0.078*	
H5C	−0.0286	0.1953	0.0880	0.078*	
C6	0.41155 (11)	0.19724 (9)	0.19625 (8)	0.0449 (3)	
C7	0.40481 (14)	0.14455 (11)	0.12339 (10)	0.0597 (4)	
H7	0.3324	0.1218	0.0894	0.072*	
C8	0.50454 (17)	0.12346 (14)	0.09794 (12)	0.0758 (5)	
H8	0.4970	0.0891	0.0467	0.091*	
C9	0.61114 (15)	0.15324 (14)	0.14808 (13)	0.0746 (5)	
H9	0.6767	0.1379	0.1315	0.090*	
C10	0.62429 (12)	0.20652 (11)	0.22432 (11)	0.0603 (4)	
C11	0.73543 (14)	0.23652 (14)	0.27924 (15)	0.0771 (5)	
H11	0.8019	0.2193	0.2646	0.093*	
C12	0.74729 (15)	0.28936 (15)	0.35201 (15)	0.0834 (6)	
H12	0.8212	0.3078	0.3870	0.100*	
C13	0.65024 (15)	0.31585 (13)	0.37427 (13)	0.0769 (5)	
H13	0.6588	0.3537	0.4237	0.092*	
C14	0.54088 (13)	0.28741 (10)	0.32481 (10)	0.0588 (4)	
H14	0.4766	0.3055	0.3419	0.071*	
C15	0.52372 (11)	0.23149 (9)	0.24881 (9)	0.0480 (3)	
C16	0.30264 (10)	0.16184 (8)	0.37488 (7)	0.0397 (2)	
C17	0.32705 (10)	0.08631 (9)	0.44286 (7)	0.0412 (3)	
C18	0.35333 (11)	0.11383 (10)	0.53084 (8)	0.0496 (3)	
H18	0.3584	0.1781	0.5453	0.060*	
C19	0.37171 (13)	0.04706 (12)	0.59598 (9)	0.0601 (4)	
H19	0.3896	0.0658	0.6545	0.072*	
C20	0.36361 (13)	−0.04786 (12)	0.57435 (10)	0.0643 (4)	
H20	0.3742	−0.0934	0.6184	0.077*	
C21	0.34002 (13)	−0.07620 (10)	0.48835 (10)	0.0574 (3)	
H21	0.3363	−0.1407	0.4749	0.069*	
C22	0.32156 (10)	−0.00945 (9)	0.42105 (8)	0.0445 (3)	
C23	0.29843 (12)	−0.03946 (8)	0.32751 (9)	0.0494 (3)	
H23A	0.2166	−0.0560	0.3029	0.059*	
H23B	0.3442	−0.0958	0.3256	0.059*	
C24	0.32839 (11)	0.03774 (8)	0.27179 (8)	0.0433 (3)	
H24A	0.3053	0.0168	0.2109	0.052*	
H24B	0.4124	0.0469	0.2907	0.052*	
C25	0.06134 (10)	0.10957 (8)	0.29967 (7)	0.0388 (2)	
C26	0.05570 (11)	0.15438 (9)	0.37485 (8)	0.0462 (3)	
H26	0.0992	0.2090	0.3950	0.055*	
C27	−0.01597 (13)	0.11681 (11)	0.42021 (9)	0.0578 (4)	
H27	−0.0198	0.1463	0.4715	0.069*	
C28	−0.08122 (13)	0.03680 (13)	0.39040 (10)	0.0634 (4)	
H28	−0.1276	0.0121	0.4224	0.076*	
C29	−0.07937 (12)	−0.00782 (11)	0.31387 (9)	0.0574 (3)	
H29	−0.1248	−0.0614	0.2929	0.069*	
C30	−0.00761 (10)	0.03006 (8)	0.26956 (8)	0.0435 (3)	
C31	0.08823 (10)	0.05013 (8)	0.16833 (8)	0.0420 (3)	
C32	−0.05995 (13)	−0.07827 (10)	0.13719 (10)	0.0569 (3)	
H32A	−0.0609	−0.1330	0.1740	0.068*	
H32B	−0.0230	−0.0974	0.0936	0.068*	
C33	−0.18359 (13)	−0.04818 (11)	0.09099 (10)	0.0604 (4)	
H33A	−0.2245	−0.1003	0.0547	0.072*	
H33B	−0.2227	−0.0349	0.1347	0.072*	

### Atomic displacement parameters (Å^2^)

**Table:**

	*U*^11^	*U*^22^	*U*^33^	*U*^12^	*U*^13^	*U*^23^
N1	0.0400 (5)	0.0397 (5)	0.0409 (5)	0.0025 (4)	0.0125 (4)	0.0074 (4)
N2	0.0489 (6)	0.0408 (5)	0.0435 (5)	−0.0068 (4)	0.0073 (4)	−0.0023 (4)
O1	0.0814 (7)	0.0387 (5)	0.0462 (5)	−0.0043 (4)	0.0181 (5)	−0.0082 (4)
O2	0.0625 (6)	0.0640 (6)	0.0414 (5)	−0.0040 (4)	0.0178 (4)	−0.0132 (4)
O3	0.0724 (7)	0.0672 (6)	0.0546 (6)	0.0100 (5)	0.0171 (5)	0.0018 (5)
C1	0.0446 (6)	0.0444 (6)	0.0425 (6)	0.0008 (5)	0.0152 (5)	0.0075 (5)
C2	0.0406 (6)	0.0385 (6)	0.0409 (6)	−0.0016 (4)	0.0131 (5)	0.0007 (4)
C3	0.0405 (6)	0.0340 (5)	0.0346 (5)	0.0005 (4)	0.0106 (4)	−0.0017 (4)
C4	0.0413 (6)	0.0353 (5)	0.0341 (5)	−0.0008 (4)	0.0113 (4)	0.0003 (4)
C5	0.0432 (7)	0.0575 (8)	0.0532 (7)	0.0032 (5)	0.0103 (5)	0.0145 (6)
C6	0.0449 (6)	0.0457 (6)	0.0461 (6)	0.0018 (5)	0.0170 (5)	0.0061 (5)
C7	0.0603 (8)	0.0694 (9)	0.0528 (7)	0.0048 (7)	0.0223 (6)	−0.0030 (6)
C8	0.0860 (12)	0.0850 (12)	0.0696 (10)	0.0159 (9)	0.0435 (9)	−0.0009 (9)
C9	0.0644 (10)	0.0850 (11)	0.0888 (12)	0.0199 (8)	0.0447 (9)	0.0227 (10)
C10	0.0489 (7)	0.0606 (8)	0.0762 (10)	0.0076 (6)	0.0264 (7)	0.0291 (7)
C11	0.0430 (8)	0.0840 (12)	0.1049 (14)	0.0056 (7)	0.0236 (8)	0.0441 (11)
C12	0.0495 (9)	0.0860 (13)	0.1007 (15)	−0.0155 (8)	0.0022 (9)	0.0262 (11)
C13	0.0629 (10)	0.0701 (10)	0.0830 (11)	−0.0189 (8)	0.0007 (8)	0.0044 (9)
C14	0.0500 (7)	0.0546 (8)	0.0660 (9)	−0.0086 (6)	0.0090 (6)	0.0026 (6)
C15	0.0430 (6)	0.0447 (6)	0.0568 (7)	0.0013 (5)	0.0159 (5)	0.0167 (5)
C16	0.0415 (6)	0.0378 (6)	0.0387 (5)	−0.0004 (4)	0.0104 (4)	−0.0034 (4)
C17	0.0386 (6)	0.0454 (6)	0.0377 (5)	0.0024 (5)	0.0088 (4)	0.0012 (5)
C18	0.0482 (7)	0.0594 (8)	0.0405 (6)	0.0038 (6)	0.0125 (5)	−0.0024 (5)
C19	0.0564 (8)	0.0838 (11)	0.0390 (6)	0.0105 (7)	0.0133 (6)	0.0085 (6)
C20	0.0606 (9)	0.0761 (10)	0.0555 (8)	0.0123 (7)	0.0167 (7)	0.0265 (7)
C21	0.0583 (8)	0.0502 (7)	0.0614 (8)	0.0060 (6)	0.0149 (6)	0.0141 (6)
C22	0.0409 (6)	0.0430 (6)	0.0469 (6)	0.0035 (5)	0.0093 (5)	0.0056 (5)
C23	0.0580 (7)	0.0339 (6)	0.0504 (7)	0.0049 (5)	0.0080 (6)	−0.0015 (5)
C24	0.0481 (6)	0.0388 (6)	0.0409 (6)	0.0058 (5)	0.0104 (5)	−0.0050 (4)
C25	0.0412 (6)	0.0378 (5)	0.0373 (5)	0.0030 (4)	0.0119 (4)	0.0058 (4)
C26	0.0499 (7)	0.0474 (6)	0.0417 (6)	0.0076 (5)	0.0147 (5)	0.0030 (5)
C27	0.0603 (8)	0.0749 (10)	0.0432 (7)	0.0141 (7)	0.0234 (6)	0.0107 (6)
C28	0.0541 (8)	0.0854 (11)	0.0550 (8)	0.0013 (7)	0.0229 (6)	0.0247 (7)
C29	0.0529 (7)	0.0608 (8)	0.0564 (8)	−0.0097 (6)	0.0134 (6)	0.0168 (6)
C30	0.0434 (6)	0.0431 (6)	0.0414 (6)	−0.0001 (5)	0.0092 (5)	0.0085 (5)
C31	0.0429 (6)	0.0414 (6)	0.0386 (6)	−0.0001 (5)	0.0079 (5)	−0.0026 (4)
C32	0.0621 (8)	0.0406 (7)	0.0592 (8)	−0.0100 (6)	0.0053 (6)	−0.0056 (6)
C33	0.0583 (8)	0.0640 (9)	0.0517 (7)	−0.0152 (6)	0.0063 (6)	−0.0021 (6)

### Geometric parameters (Å, º)

**Table:**

N1—C5	1.4545 (16)	C12—H12	0.9300
N1—C1	1.4582 (15)	C13—C14	1.370 (2)
N1—C4	1.4681 (14)	C13—H13	0.9300
N2—C31	1.3522 (16)	C14—C15	1.406 (2)
N2—C30	1.3996 (16)	C14—H14	0.9300
N2—C32	1.4508 (16)	C16—C17	1.4812 (16)
O1—C16	1.2091 (14)	C17—C22	1.3865 (17)
O2—C31	1.2154 (15)	C17—C18	1.3957 (16)
O3—C33	1.3979 (18)	C18—C19	1.3669 (19)
O3—H3	0.8200	C18—H18	0.9300
C1—C2	1.5222 (16)	C19—C20	1.374 (2)
C1—H1A	0.9700	C19—H19	0.9300
C1—H1B	0.9700	C20—C21	1.372 (2)
C2—C6	1.5069 (17)	C20—H20	0.9300
C2—C3	1.5729 (15)	C21—C22	1.3914 (18)
C2—H2	0.9800	C21—H21	0.9300
C3—C24	1.5308 (15)	C22—C23	1.4926 (18)
C3—C16	1.5423 (15)	C23—C24	1.5142 (18)
C3—C4	1.5828 (16)	C23—H23A	0.9700
C4—C25	1.5118 (15)	C23—H23B	0.9700
C4—C31	1.5572 (15)	C24—H24A	0.9700
C5—H5A	0.9600	C24—H24B	0.9700
C5—H5B	0.9600	C25—C26	1.3743 (17)
C5—H5C	0.9600	C25—C30	1.3868 (17)
C6—C7	1.3593 (19)	C26—C27	1.3876 (19)
C6—C15	1.4361 (18)	C26—H26	0.9300
C7—C8	1.410 (2)	C27—C28	1.370 (2)
C7—H7	0.9300	C27—H27	0.9300
C8—C9	1.353 (3)	C28—C29	1.378 (2)
C8—H8	0.9300	C28—H28	0.9300
C9—C10	1.395 (3)	C29—C30	1.3784 (18)
C9—H9	0.9300	C29—H29	0.9300
C10—C11	1.420 (2)	C32—C33	1.504 (2)
C10—C15	1.4236 (19)	C32—H32A	0.9700
C11—C12	1.348 (3)	C32—H32B	0.9700
C11—H11	0.9300	C33—H33A	0.9700
C12—C13	1.371 (3)	C33—H33B	0.9700
			
C5—N1—C1	114.25 (9)	O1—C16—C17	120.16 (10)
C5—N1—C4	115.50 (9)	O1—C16—C3	120.81 (10)
C1—N1—C4	105.70 (9)	C17—C16—C3	119.02 (9)
C31—N2—C30	111.13 (10)	C22—C17—C18	120.00 (11)
C31—N2—C32	124.16 (11)	C22—C17—C16	121.90 (10)
C30—N2—C32	124.42 (11)	C18—C17—C16	118.09 (11)
C33—O3—H3	109.5	C19—C18—C17	120.55 (13)
N1—C1—C2	102.12 (9)	C19—C18—H18	119.7
N1—C1—H1A	111.3	C17—C18—H18	119.7
C2—C1—H1A	111.3	C18—C19—C20	119.55 (13)
N1—C1—H1B	111.3	C18—C19—H19	120.2
C2—C1—H1B	111.3	C20—C19—H19	120.2
H1A—C1—H1B	109.2	C21—C20—C19	120.66 (13)
C6—C2—C1	117.06 (10)	C21—C20—H20	119.7
C6—C2—C3	114.98 (9)	C19—C20—H20	119.7
C1—C2—C3	104.82 (9)	C20—C21—C22	120.73 (14)
C6—C2—H2	106.4	C20—C21—H21	119.6
C1—C2—H2	106.4	C22—C21—H21	119.6
C3—C2—H2	106.4	C17—C22—C21	118.48 (12)
C24—C3—C16	107.63 (9)	C17—C22—C23	120.34 (11)
C24—C3—C2	114.47 (9)	C21—C22—C23	121.17 (12)
C16—C3—C2	108.55 (9)	C22—C23—C24	112.28 (10)
C24—C3—C4	114.06 (9)	C22—C23—H23A	109.1
C16—C3—C4	108.89 (9)	C24—C23—H23A	109.1
C2—C3—C4	103.02 (8)	C22—C23—H23B	109.1
N1—C4—C25	112.58 (9)	C24—C23—H23B	109.1
N1—C4—C31	110.16 (9)	H23A—C23—H23B	107.9
C25—C4—C31	100.96 (9)	C23—C24—C3	113.42 (10)
N1—C4—C3	103.03 (8)	C23—C24—H24A	108.9
C25—C4—C3	118.06 (9)	C3—C24—H24A	108.9
C31—C4—C3	112.22 (9)	C23—C24—H24B	108.9
N1—C5—H5A	109.5	C3—C24—H24B	108.9
N1—C5—H5B	109.5	H24A—C24—H24B	107.7
H5A—C5—H5B	109.5	C26—C25—C30	119.19 (11)
N1—C5—H5C	109.5	C26—C25—C4	131.81 (11)
H5A—C5—H5C	109.5	C30—C25—C4	109.00 (10)
H5B—C5—H5C	109.5	C25—C26—C27	118.99 (13)
C7—C6—C15	118.79 (12)	C25—C26—H26	120.5
C7—C6—C2	121.02 (12)	C27—C26—H26	120.5
C15—C6—C2	120.13 (11)	C28—C27—C26	120.81 (13)
C6—C7—C8	121.79 (15)	C28—C27—H27	119.6
C6—C7—H7	119.1	C26—C27—H27	119.6
C8—C7—H7	119.1	C27—C28—C29	121.20 (13)
C9—C8—C7	120.01 (16)	C27—C28—H28	119.4
C9—C8—H8	120.0	C29—C28—H28	119.4
C7—C8—H8	120.0	C30—C29—C28	117.41 (13)
C8—C9—C10	121.04 (14)	C30—C29—H29	121.3
C8—C9—H9	119.5	C28—C29—H29	121.3
C10—C9—H9	119.5	C29—C30—C25	122.36 (12)
C9—C10—C11	122.12 (15)	C29—C30—N2	127.39 (12)
C9—C10—C15	119.47 (14)	C25—C30—N2	110.25 (10)
C11—C10—C15	118.41 (17)	O2—C31—N2	125.07 (11)
C12—C11—C10	121.79 (16)	O2—C31—C4	126.21 (11)
C12—C11—H11	119.1	N2—C31—C4	108.64 (10)
C10—C11—H11	119.1	N2—C32—C33	112.50 (12)
C11—C12—C13	119.87 (16)	N2—C32—H32A	109.1
C11—C12—H12	120.1	C33—C32—H32A	109.1
C13—C12—H12	120.1	N2—C32—H32B	109.1
C12—C13—C14	120.98 (19)	C33—C32—H32B	109.1
C12—C13—H13	119.5	H32A—C32—H32B	107.8
C14—C13—H13	119.5	O3—C33—C32	113.01 (12)
C13—C14—C15	121.44 (16)	O3—C33—H33A	109.0
C13—C14—H14	119.3	C32—C33—H33A	109.0
C15—C14—H14	119.3	O3—C33—H33B	109.0
C14—C15—C10	117.47 (13)	C32—C33—H33B	109.0
C14—C15—C6	123.69 (12)	H33A—C33—H33B	107.8
C10—C15—C6	118.82 (13)		
			
C5—N1—C1—C2	−175.59 (10)	O1—C16—C17—C22	179.21 (12)
C4—N1—C1—C2	−47.49 (11)	C3—C16—C17—C22	0.48 (16)
N1—C1—C2—C6	162.32 (10)	O1—C16—C17—C18	0.81 (17)
N1—C1—C2—C3	33.58 (11)	C3—C16—C17—C18	−177.92 (10)
C6—C2—C3—C24	−14.77 (14)	C22—C17—C18—C19	−1.21 (19)
C1—C2—C3—C24	115.21 (10)	C16—C17—C18—C19	177.22 (12)
C6—C2—C3—C16	105.48 (11)	C17—C18—C19—C20	−0.3 (2)
C1—C2—C3—C16	−124.54 (10)	C18—C19—C20—C21	1.6 (2)
C6—C2—C3—C4	−139.17 (10)	C19—C20—C21—C22	−1.3 (2)
C1—C2—C3—C4	−9.19 (11)	C18—C17—C22—C21	1.49 (18)
C5—N1—C4—C25	−63.23 (13)	C16—C17—C22—C21	−176.88 (11)
C1—N1—C4—C25	169.41 (9)	C18—C17—C22—C23	−177.44 (11)
C5—N1—C4—C31	48.61 (13)	C16—C17—C22—C23	4.19 (18)
C1—N1—C4—C31	−78.75 (11)	C20—C21—C22—C17	−0.3 (2)
C5—N1—C4—C3	168.51 (10)	C20—C21—C22—C23	178.64 (13)
C1—N1—C4—C3	41.16 (10)	C17—C22—C23—C24	21.81 (17)
C24—C3—C4—N1	−142.90 (9)	C21—C22—C23—C24	−157.09 (12)
C16—C3—C4—N1	96.87 (10)	C22—C23—C24—C3	−53.54 (14)
C2—C3—C4—N1	−18.23 (10)	C16—C3—C24—C23	55.80 (13)
C24—C3—C4—C25	92.34 (12)	C2—C3—C24—C23	176.56 (10)
C16—C3—C4—C25	−27.88 (13)	C4—C3—C24—C23	−65.12 (13)
C2—C3—C4—C25	−142.98 (9)	N1—C4—C25—C26	−61.43 (16)
C24—C3—C4—C31	−24.43 (13)	C31—C4—C25—C26	−178.86 (12)
C16—C3—C4—C31	−144.66 (9)	C3—C4—C25—C26	58.47 (16)
C2—C3—C4—C31	100.24 (10)	N1—C4—C25—C30	117.98 (10)
C1—C2—C6—C7	−42.55 (17)	C31—C4—C25—C30	0.55 (11)
C3—C2—C6—C7	81.16 (15)	C3—C4—C25—C30	−122.12 (10)
C1—C2—C6—C15	140.16 (12)	C30—C25—C26—C27	2.18 (17)
C3—C2—C6—C15	−96.14 (13)	C4—C25—C26—C27	−178.46 (12)
C15—C6—C7—C8	−0.5 (2)	C25—C26—C27—C28	−0.6 (2)
C2—C6—C7—C8	−177.84 (14)	C26—C27—C28—C29	−1.2 (2)
C6—C7—C8—C9	2.2 (3)	C27—C28—C29—C30	1.3 (2)
C7—C8—C9—C10	−1.3 (3)	C28—C29—C30—C25	0.3 (2)
C8—C9—C10—C11	178.36 (16)	C28—C29—C30—N2	179.84 (13)
C8—C9—C10—C15	−1.2 (2)	C26—C25—C30—C29	−2.08 (18)
C9—C10—C11—C12	178.86 (16)	C4—C25—C30—C29	178.42 (11)
C15—C10—C11—C12	−1.6 (2)	C26—C25—C30—N2	178.34 (10)
C10—C11—C12—C13	−0.3 (3)	C4—C25—C30—N2	−1.16 (13)
C11—C12—C13—C14	1.7 (3)	C31—N2—C30—C29	−178.19 (13)
C12—C13—C14—C15	−1.1 (3)	C32—N2—C30—C29	7.8 (2)
C13—C14—C15—C10	−0.9 (2)	C31—N2—C30—C25	1.36 (14)
C13—C14—C15—C6	177.94 (14)	C32—N2—C30—C25	−172.66 (11)
C9—C10—C15—C14	−178.30 (13)	C30—N2—C31—O2	−177.80 (12)
C11—C10—C15—C14	2.11 (19)	C32—N2—C31—O2	−3.8 (2)
C9—C10—C15—C6	2.82 (19)	C30—N2—C31—C4	−0.96 (13)
C11—C10—C15—C6	−176.76 (12)	C32—N2—C31—C4	173.07 (11)
C7—C6—C15—C14	179.24 (13)	N1—C4—C31—O2	57.84 (15)
C2—C6—C15—C14	−3.40 (18)	C25—C4—C31—O2	177.03 (12)
C7—C6—C15—C10	−1.96 (18)	C3—C4—C31—O2	−56.34 (15)
C2—C6—C15—C10	175.39 (11)	N1—C4—C31—N2	−118.95 (10)
C24—C3—C16—O1	151.83 (12)	C25—C4—C31—N2	0.25 (11)
C2—C3—C16—O1	27.41 (15)	C3—C4—C31—N2	126.88 (10)
C4—C3—C16—O1	−84.06 (13)	C31—N2—C32—C33	−103.09 (15)
C24—C3—C16—C17	−29.45 (13)	C30—N2—C32—C33	70.17 (16)
C2—C3—C16—C17	−153.87 (10)	N2—C32—C33—O3	57.18 (17)
C4—C3—C16—C17	94.66 (11)		

### Hydrogen-bond geometry (Å, º)

Cg1 is the centroid of the C10–C15 phenyl ring.

**Table:**

*D*—H···*A*	*D*—H	H···*A*	*D*···*A*	*D*—H···*A*
C26—H26···O1	0.93	2.54	3.198 (2)	128
C24—H24*A*···O2	0.97	2.42	3.098 (2)	127
C14—H14···O1	0.93	2.59	3.394 (2)	145
C2—H2···O1	0.98	2.21	2.764 (2)	115
C1—H1*B*···O2	0.97	2.40	3.020 (2)	121
O3—H3···O2^i^	0.82	2.03	2.830 (1)	164
C20—H20···*Cg*1^ii^	0.93	2.71	3.603 (2)	161

Symmetry codes: (i) −*x*, −*y*, −*z*; (ii) −*x*, −*y*, −*z*+1.
